# The most efficient machine learning algorithms in stroke prediction: A systematic review

**DOI:** 10.1002/hsr2.70062

**Published:** 2024-10-01

**Authors:** Farkhondeh Asadi, Milad Rahimi, Amir Hossein Daeechini, Atefeh Paghe

**Affiliations:** ^1^ Department of Health Information Technology and Management School of Allied Medical Sciences, Shahid Beheshti University of Medical Sciences Tehran Iran; ^2^ Department of Health Information Technology Urmia University of Medical Sciences Urmia Iran

**Keywords:** artificial intelligence, machine learning, prediction, stroke

## Abstract

**Background and Aims:**

Stroke is one of the most common causes of death worldwide, leading to numerous complications and significantly diminishing the quality of life for those affected. The purpose of this study is to systematically review published papers on stroke prediction using machine learning algorithms and introduce the most efficient machine learning algorithms and compare their performance. The papers have published in period from 2019 to August 2023.

**Methods:**

The authors conducted a systematic search in PubMed, Scopus, Web of Science, and IEEE using the keywords “Artificial Intelligence,” “Predictive Modeling,” “Machine Learning,” “Stroke,” and “Cerebrovascular Accident” from 2019 to August 2023.

**Results:**

Twenty articles were included based on the inclusion criteria. The Random Forest (RF) algorithm was introduced as the best and most efficient stroke ML algorithm in 25% of the articles (*n* = 5). In addition, in other articles, Support Vector Machines (SVM), Stacking and XGBOOST, DSGD, COX& GBT, ANN, NB, and RXLM algorithms were introduced as the best and most efficient ML algorithms in stroke prediction.

**Conclusion:**

This research has shown a rapid increase in using ML algorithms to predict stroke, with significant improvements in model accuracy in recent years. However, no model has reached 100% accuracy or is entirely error‐free. Variations in algorithm efficiency and accuracy stem from differences in sample sizes, datasets, and data types. Further studies should focus on consistent datasets, sample sizes, and data types for more reliable outcomes.

## 1 INTRODUCTION

Stroke, a leading neurological disorder worldwide, is responsible for over 12.2 million new cases each year. It primarily occurs when the brain's blood supply is disrupted by blood clots, blocking blood flow, or when blood vessels rupture, causing bleeding and damage to brain tissue.^1^, ^2^, ^3^ Deprivation of cells from oxygen and other nutrients during a stroke leads to the death of cells, ending in permanent disability and even death.^4^

Ischemic and hemorrhagic stroke are two types of strokes. In ischemic stroke, the blood clot blocks the cerebral vessels, and in hemorrhagic stroke, bleeding occurs inside the brain.^5^, ^6^ High blood pressure, obesity, physical inactivity, and smoking are among the most critical risk factors in stroke sufferers.^7^

Experts predict that both the death rate from strokes and the number of people affected by this condition will rise alongside global population growth. However, these fatalities can be significantly reduced through early detection and treatment.^8^

In the past decades, with the emergence of evidence‐based approaches to stroke prevention, acute stroke management, and stroke recovery, there has been a significant shift in the field of stroke, and the mission of the World Stroke Organization (WSO) is to reduce the global burden of stroke through prevention, treatment, and long‐term care.^9^

Today, technology's role in healthcare has grown significantly, capturing the interest of medical professionals. Machine Learning (ML) algorithms, in particular, are being leveraged to produce precise data for diagnosing, prognosis, and predicting various diseases.^10^, ^11^, ^12^ Furthermore, developing predictive tools powered by Artificial Intelligence (AI) can potentially prevent and decrease stroke occurrences.^13^ As a branch of AI, ML offers an array of models capable of detecting intricate patterns, understanding the connections among them, and utilizing this knowledge for prediction or decision‐making purposes.^14^, ^15^, ^16^

AI techniques differ from traditional prediction models because they utilize and combine a vast array of variables to describe the ambiguous and complex nature of human physiology.^5^ In addition to helping with prevention, diagnosis, and patient monitoring, they can play a crucial role in managing a vast amount of data accurately and practically.^17^

AI algorithms can accurately classify, diagnose, and segment the lesions in the brain tissue. With the help of AI, doctors can diagnose intracranial bleeding, microbial bleeding, and acute ischemic stroke more efficiently.^18^ AI uses unique ML algorithms to “learn” features from large data sets and recognize patterns that are often invisible to the human eye.^13^

ML technology employs a range of techniques for automated data analysis, including linear and logistic regression models, Support Vector Machines (SVM), Random Forests (RF), classification trees, and discriminant analysis.^19^, ^20^ ML is a multivariate approach that can be used to analyze complex and heterogeneous data types and include them in risk prediction models, making it a promising solution for stroke risk prediction.^13^

The purpose of this study is to systematically review published papers on stroke prediction using machine learning algorithms and introduce the most efficient machine learning algorithms and compare their performance. The papers have published in period from 2019 to August 2023.

## 2 METHODS

The authors conducted a thorough search following specific inclusion criteria they developed. All articles that used ML to predict the occurrence of stroke were reviewed. A comprehensive search was conducted from 2019 to August 2023 using selected keywords in PubMed, Scopus, Web of Science, and IEEE databases. The search strategy is illustrated in Table 1.

**Table 1:** The strategy of systematic search in four defined databases.

#A	“Stroke” OR “Cerebrovascular Accident”
#B	“Machine Learning,” OR “Artificial Intelligence,” OR “predictive modeling”
Strategy	(#A) AND (#B)

### 2.1 Eligibility of articles

**Figure 1:**
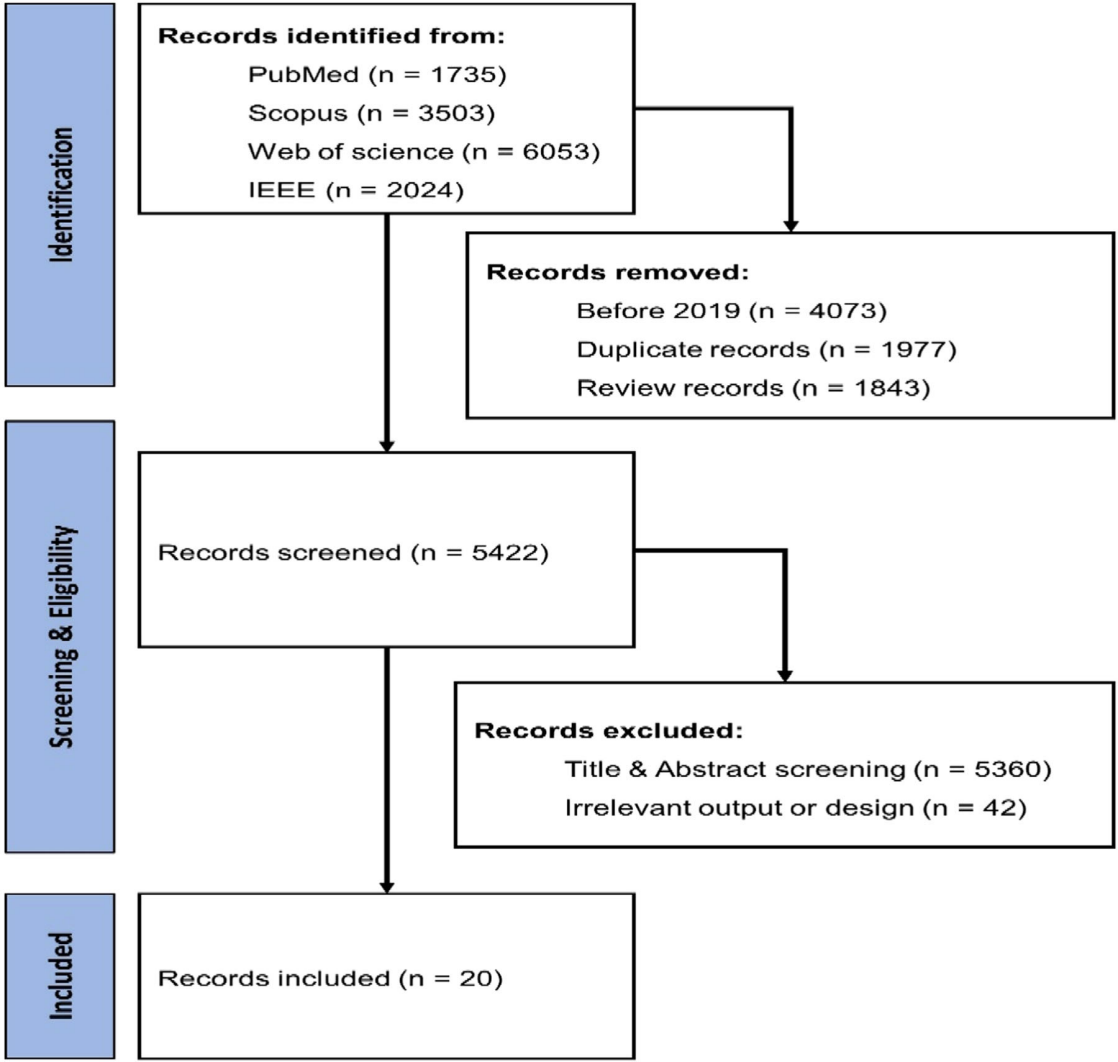
PRISMA flow diagram for study identification and selection process.

Specific criteria were defined for the inclusion and exclusion of articles. Inclusion criteria were articles that used ML algorithms to predict stroke, articles written in English, available full‐text articles, and articles published between 2019 and August 2023. Articles related to other diseases, articles published in languages other than English, articles that used deep learning algorithms, review articles, meta‐analyses, books, letters to the editor, or conference papers were excluded from the systematic review. The selection process of articles is depicted in the PRISMA flow diagram Figure 1.

In this systematic review, the data extraction process included a thorough review of previous articles to gather information about their methods and results. Articles were extracted using standardized table formats. Extracted information was the first author of the article, the countries included in the study, the year of publication of the article, the characteristics and dimensions of the studied data set, the types of ML algorithms used and the best algorithm, the evaluation criteria of ML algorithms and selected features in stroke prediction.

### 2.2 Evaluation criteria of models

The formula for the Computation of the performance evaluation parameters of the models is given below.

1. **Accuracy:**

(1) $$\text{Accuracy}\;=\frac{\mathrm{TP}+\mathrm{TN}}{\mathrm{TP}+\mathrm{FP}+\mathrm{FN}+\mathrm{TN}}$$

− The ratio of correctly predicted instances to the total instances. It is calculated as:
− Where:

- (TP) = True Positives: predicted to be positive and the actual value is also positive
- (TN) = True Negatives: predicted to be negative and the actual value is also negative
- (FP) = False Positives: predicted to be positive but the actual value is negative
- (FN) = False Negatives: predicted to be negative but the actual value is positive

2. **Precision:**

− The ratio of correctly predicted positive observations to the total predicted positives. It is calculated as:

(2) $$\text{Precision}\;=\frac{\text{TP}}{\text{TP}+\text{FP}}$$

3. **Recall (Sensitivity or True Positive Rate):**

− The ratio of correctly predicted positive observations to all observations in the actual class. It is calculated as:

(3) $$\text{Recall}\;=\frac{\text{TP}}{\text{TP}+\text{FN}}$$

4. **F1 Score:**

− The harmonic means of precision and recall, providing a balance between the two. It is calculated as:

(4) $$F1-\text{score}=\frac{2*\text{recall}*\text{Precision}}{\text{precision}+\text{recall}}$$

5. **Specificity (True Negative Rate):**

− The ratio of correctly predicted negative observations to all actual negatives. It is calculated as:

(5) $$\text{Specificity}\;=\frac{\mathrm{TN}}{\mathrm{TN}+\mathrm{FP}}$$

6. **Area Under the Receiver Operating Characteristic Curve (AUC‐ROC):**

− AUC‐ROC measures the model's ability to distinguish between classes. The ROC curve plots the true positive rate (sensitivity) against the false positive rate (1 ‐ specificity). The AUC represents the degree or measure of separability.

7. **Positive Predictive Value (PPV):**

− Synonymous with precision, it is the probability that subjects with a positive screening test truly have the disease.

8. **Negative Predictive Value (NPV):**

− The ratio of true negative observations to the total predicted negatives. It is calculated as:

(6) $$\text{NPV}\;=\frac{\mathrm{TN}}{\mathrm{FN}+\mathrm{TN}}$$

9. **Kappa (Cohen's Kappa):**

− A statistic that measures inter‐rater agreement for categorical items. It accounts for the agreement occurring by chance. It is calculated as:

(7) $$\kappa =\frac{P_{o}-P_{e}}{1-P_{e}}$$

− Where:
− (P_o_) = Observed Agreement
− (P_e_) = Expected Agreement

10. **Matthews Correlation Coefficient (MCC):**

− A balanced measure that takes into account true and false positives and negatives, useful for binary classification even if the classes are of very different sizes. It is calculated as:

(8) $$\text{MCC}\;=\;\frac{\left(\mathrm{TP}\;\times \;\mathrm{TN}\right)\;-\;\left(\mathrm{FP}\;\times \;\mathrm{FN}\right)}{\sqrt{\left(\mathrm{TP}\;+\;\mathrm{FP}\right)\left(\mathrm{TP}\;+\;\mathrm{FN}\right)\left(\mathrm{TN}\;+\;\mathrm{FP}\right)\left(\mathrm{TN}\;+\;\mathrm{FN}\right)}}$$

These criteria help evaluate different aspects of the model's performance, ensuring a comprehensive assessment beyond mere accuracy.

## 3 RESULTS

In the systematic search to identify articles related to stroke prediction with ML algorithms, 5422 articles were identified in the first step. Then, 5360 articles were screened based on the title and abstract, and 42 other articles were removed based on the full text. Ultimately, 20 full‐text articles were included in the final analysis (Table 2).

**Table 2:** The characteristics of the included studies.

Ref	First Author	Journal	Countries	Publication year	Data set (Records ‐ Variables)	Data types	Balancing technique	(*) =Best model
[21]	Krishna Mridha	IEEE Access	India Japan Bangladesh	2023	Kaggle (5110 ‐ 12)	clinical	SMOTE	*RF
[22]	Dritsas	Sensors (Basel)	Greece	2022	Kaggle (3254 ‐ 11)	clinical	SMOTE	*Stacking
[23]	Asmir Vodencarevic	Stroke	Germany Canada	2022	Erlangen Stroke Registry (ESPro) (384 ‐ 250)	Demographics Comorbidities Interactions Clinical	SMOTE/ under‐sampling /COST/ Anomaly Detection Techniques	*SVM
[24]	Matthew Chun	JAMIA	China UK	2021	participants enrolled from 10 geographically diverse areas of China (512726 ‐ 143)	Sociodemographic Lifestyle Clinical	Ensemble Methods	*COX *GBT
[25]	Eman M Alanazi	JMIR FORMATIVE RESEARCH	United States Saudi Arabia Egypt	2021	National Health and Nutrition Examination Survey (NHANES) (4186 ‐ 21)	Demographics Dietary Examination Laboratory questioner	Data resampling	*RF
[26]	Yujie Yang	JMIR Med Inform	China	2021	EHRs from the Shenzhen Health Information (57671 ‐ 49)	Lifestyle Demographics Family history medical history Physical exam	‐	*XGBoost
[27]	Nojood Alageel	IJACSA	Saudi Arabia	2023	Kaggle (3254 ‐ 9)	Clinical	SMOTE	*Stacking
[8]	Biswas	Healthcare Analytics	Bangladesh	2022	Kaggle (43400 ‐ 12)	Clinical	Random Over Sampling	*SVM
[28]	Samaa A. Mostafa	IJACSA	Egypt	2022	Kaggle (5110 ‐ 12)	Clinical	SMOTE	*Stacking
[29]	Vamsi Bandi	IIETA	Malaysia India	2020	medical records based on NIHSS (4799 ‐ 10)	Clinical	‐	*RF
[30]	Vinay Padimi	ETRI journal	India USA	2022	(196102 ‐ 23)	Clinical	under‐sampling	*RF
[31]	Meshrif Alruily	Applied science	Saudi Arabia	2023	Kaggle (5110 ‐ 11)	Clinical	SMOTE	*RXLM model (RF/XGBoost & LightGBM)
[32]	Tahia Tazin	Journal of Healthcare Engineering	Bangladesh Saudi Arabia	2021	Kaggle (5110 ‐ 12)	Clinical	SMOTE	*RF
[33]	Nwosu	Annu Int Conf IEEE Eng Med Biol Soc	Ireland Singapore	2019	Kaggle (29072 ‐ 12)	Clinical	under‐sampling	*ANN
[34]	Gangavarapu Sailasya	IJACSA	India	2021	Kaggle (5110 ‐ 12)	Clinical	under sampling	*NB
[35]	Kazutaka Uchida	Translational Stroke Research	Japan	2022	patient in three cities in Japan (3178 ‐ 19)	Clinical	‐	LR RF XGBoost
[36]	Qi Wang	Frontiers in Aging Neuroscience	China	2022	participants were from the community‐dwelling population in Suzhou (4503 ‐ 38)	Lifestyle Clinical Demographics	‐	LR
[37]	Xiao Zhang	Frontiers in Aging Neuroscience	China	2022	MIMIC ‐III MIMIC‐VI databases (7789 ‐ 51)	Clinical Demographics	SMOTE	*XGBoost
[38]	Fadratul Hafinaz Hassan	Baghdad Science Journal	Malaysia	2021	Kaggle (‐ 8)	Clinical	‐	*ANN
[39]	SERGIO PEÑAFIEL	IEEE Access	Chile Japan	2020	(EHR) Okayama hospital Japan (27876 ‐)	Demographics Patient history	‐	*DSGD

Abbreviations: AUC, Area Under the Curve; ANN, Artificial Neural Network; BN, Bayesian Network; BRL, Bayesian Rule List; DT, Decision tree; EHR, Electronic Health Record; GBT, Gradient boosting; KNN, K‐Nearest Neighbor; LR, Logistic Regression; ML, Machine Learning; MLP, Multilayer Perceptron Network; NB, Naïve Bayes; NPV, Negative Predictive Value; PPV, Positive Predictive Value; Smote, synthetic minority oversampling technique; SVM, Support Vector Machines; SGD, Stochastic gradient descent; XGB, eXtreme Gradient Boosting.

### 3.1 Study features

**Figure 2:**
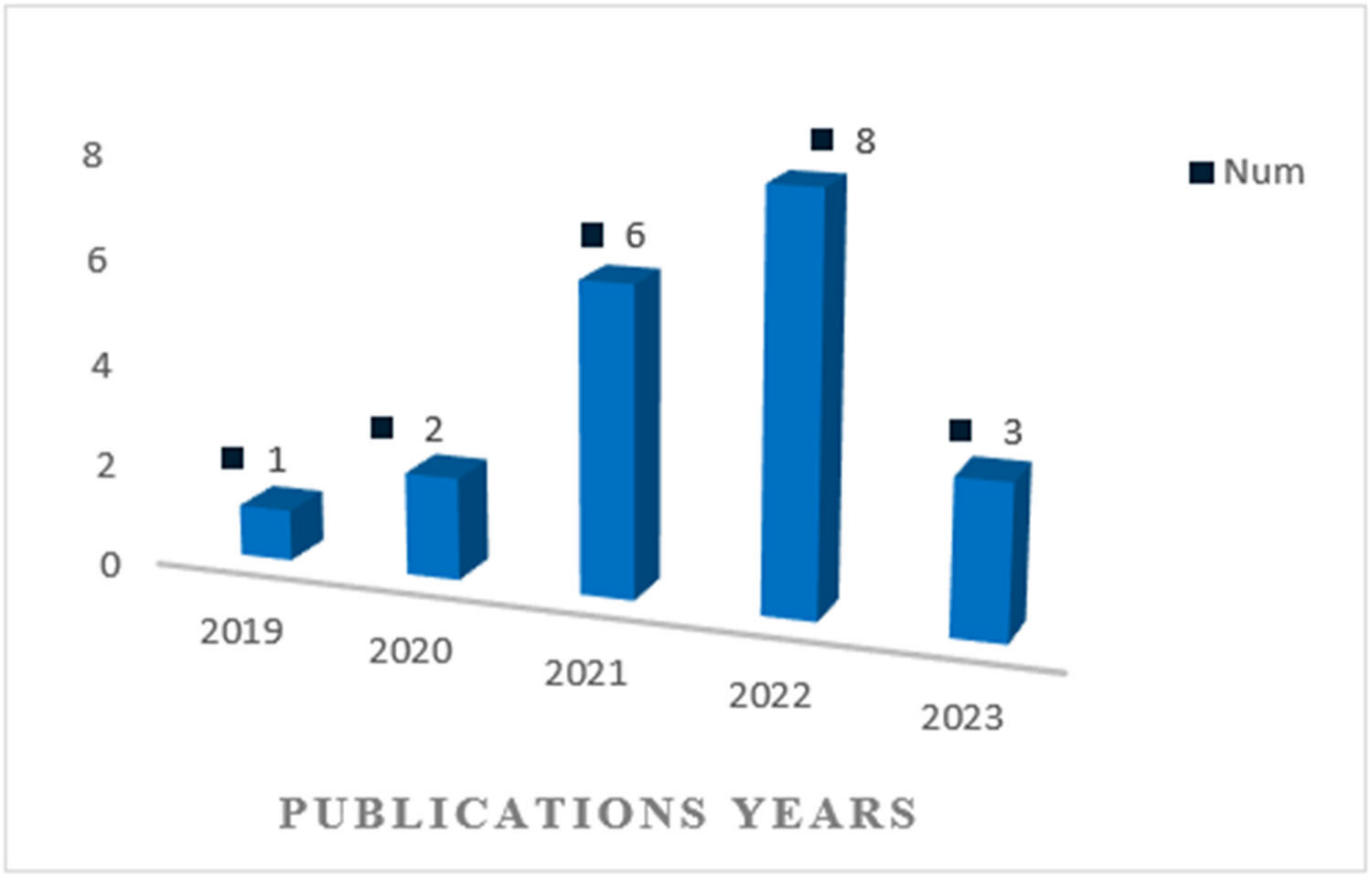
The number of published articles predicting stroke using ML algorithms from 2019 to August 2023.

**Figure 3:**
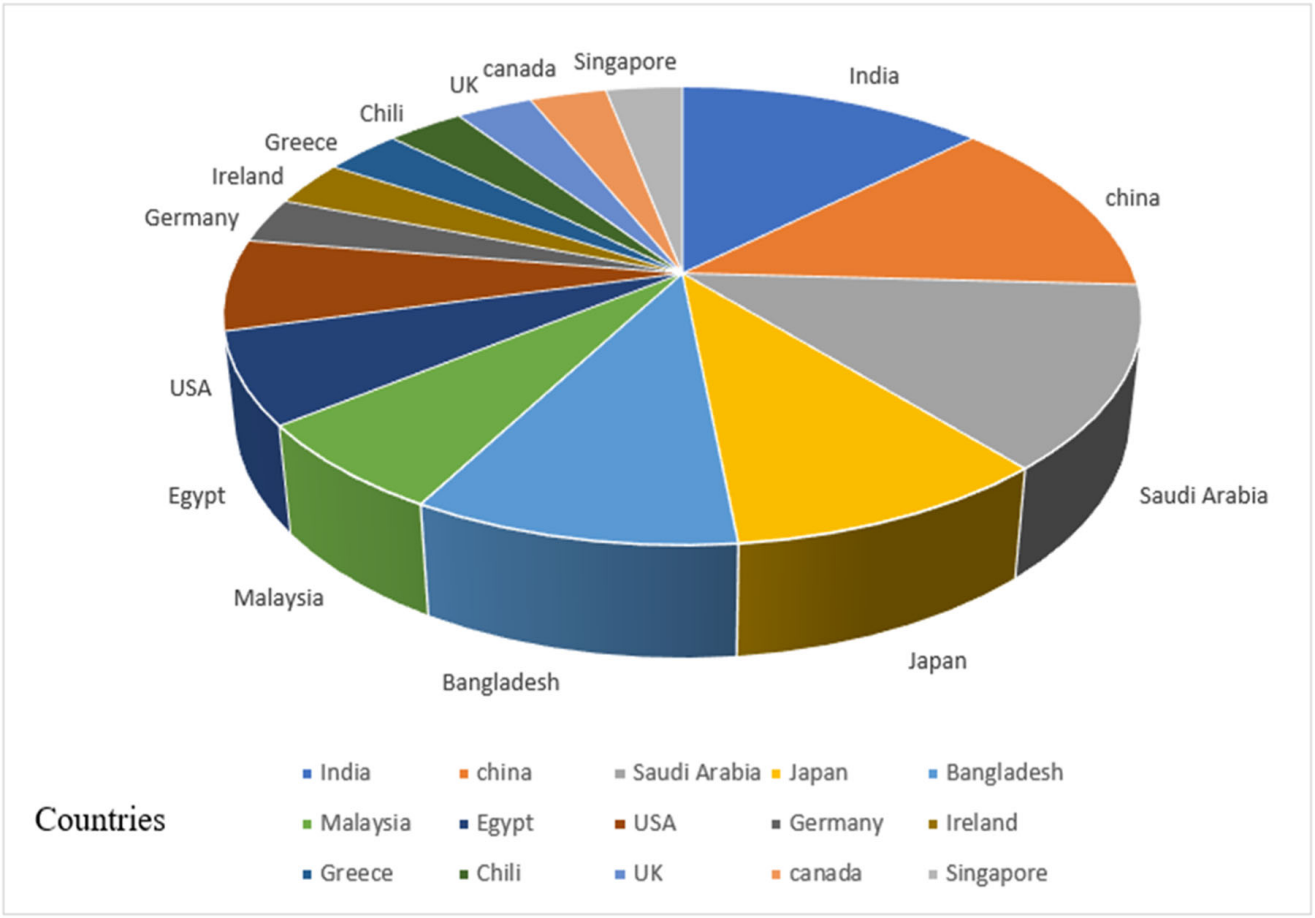
Geographic distribution of published stroke prediction studies (2019–2023).

All articles included in this study date from 2019 onward (Figure 2). These studies have been conducted in different geographical areas, including four studies in China,^24^, ^26^, ^36^, ^37^ four in India,^21^, ^29^, ^30^, ^34^ four in Saudi Arabia,^25^, ^27^, ^31^, ^32^ three in Japan,^21^, ^35^, ^39^ three in Bangladesh,^8^, ^21^, ^32^ two in Malaysia,^29^, ^38^ two in Egypt,^25^, ^28^ two in the United States,^25^, ^30^ one in Greece,^22^ one in Ireland,^33^ one in Germany,^23^ one in Chile,^39^ one in England,^24^ one in Canada ^23^ and one in Singapore ^33^ (Figure 3). Among these studies, one article was conducted in the form of a prospective cohort study on half a million Chinese adults,^24^ and the other article was conducted in the form of a 2‐year longitudinal cohort study in Southeast China.^36^ Another study developed a web page and mobile application to improve the display of results.^8^

### 3.2 Datasets characteristics

Twenty‐eight articles were excluded from the research because they concentrated on clinical aspects that needed clear guidance on implementing the ML algorithm. Among the imported articles, 10 are from Kaggle data,^8^, ^21^, ^22^, ^27^, ^28^, ^31^, ^32^, ^33^, ^34^, ^38^ three are from Electronic Health Records (EHR) ^26^, ^29^, ^39^ and two used data from disease registry centers.^23^, ^25^ Additionally, one article used MIMIC‐III and MIMIC‐VI databases to support research in intelligent patient monitoring.^37^

In most of the articles, clinical data are presented, and in several articles, in addition to clinical data, other data such as demographic and lifestyle data were used.^23^, ^24^, ^25^, ^26^, ^36^, ^37^, ^39^ Among the articles that had complete information on the data set used, the maximum and minimum sizes of the data set used for modeling were 512,726 and 384 records, respectively,^23^, ^24^ and only in one article were the datasets less than 1000 records.^23^ This diversity in data set sizes and types underscores the varied approaches to ML‐based stroke prediction in current research.

### 3.3 Significant features

The analysis of significant features extracted from various studies highlights several key factors that influence stroke prediction (Table 3). Age emerged as a consistently significant feature across multiple studies, underscoring its critical role in stroke risk assessment. Other important features include glucose level, blood pressure, and gender, which were identified in several studies as influential in predicting stroke. Additional factors such as marital status, medical history, comorbidities, and lifestyle choices like exercise and Smoking status play a crucial role. Biomarkers such as hematocrit, lymphocyte percentage, neutrophils, serum folate, hemoglobin, and homocysteine levels further contribute to stroke prediction models. These findings suggest that a multifaceted approach, incorporating demographic, clinical, and lifestyle variables, is essential for accurate stroke prediction and effective early intervention strategies.

**Table 3:** Significant features stroke prediction data set.

NO.	Significant feature	Range	Ref.
1	Age	Float	[8, 21, 22, 23, 24, 25, 26, 27, 28, 30, 31, 32, 33, 34, 35, 36, 37, 38, 39]
2	Gender	Male–Female	[8, 21, 22, 25, 26, 27, 28, 30, 31, 32, 33, 34, 35, 36, 37]
3	Ever Married	Yes–No	[8, 21, 22, 27, 28, 31, 32, 33, 34]
4	Work Type	Never_worked/Children/Private/ Self‐employed/or Govt_job	[8, 21, 22, 27, 28, 31, 33, 34]
5	Residence type	Urban‐Rural	[8, 21, 22, 27, 28, 31, 33, 34]
6	Average glucose level	Float	[8, 21, 22, 27, 28, 29, 31, 32, 33, 34, 36, 39]
7	Smoking Status	Never smoked/smoked/or Formerly Smoked	[8, 21, 22, 24, 26, 27, 28, 29, 30, 31, 33, 34, 36, 38]
8	BMI	Float	[8, 21, 22, 27, 28, 29, 31, 32, 33, 34, 36, 37, 39]
9	Hypertension	0 = No Hypertension 1 =Hypertension	[8, 21, 22, 26, 27, 28, 31, 32, 33, 34, 36, 37]
10	Heart Disease	0 = No Heart Disease 1 = heart disease	[8, 21, 22, 24, 27, 28, 30, 31, 32, 33, 34, 38]
11	STROKE	0 = No Stroke 1 = Stroke	[21, 27, 28, 31, 34]
12	ID	Float	[28, 34]
13	Biomarkers	Float	[25, 29, 36, 37, 38, 39]
14	Systolic & Diastolic BP	Float	[23, 24, 26, 29, 30, 35, 36, 37]
15	Cholesterol	0 = No Cholesterol 1 = Cholesterol	[29]
16	Paralysis	0 = No Paralysis 1 = Paralysis	[29, 35]
17	Hyperlipidemia	0 = No Hyperlipidemia 1 = Hyperlipidemia	[36, 38]
18	Diabetes	0 = No Diabetes 1 = Diabetes	[24, 26, 30, 36, 37]
19	Medical history	Yes–No	[26, 36]
20	Cancer	0 = No Cancer 1 = Cancer	[30, 37]
21	Obesity	0 = No Obesity 1 = Obesity	[30]
22	Race	Asian/Black/White/Other	[37]
23	Waist Measurement	Float	[36, 39]
24	Percutaneous endoscopic gastrotomy	Yes–No	[23]
25	Meat and vegetarian	balanced/more meat/vegetarian based	[36]
26	Arrhythmia	Yes–No	[35]
27	Nausea or vomiting,	Yes–No	[35]
28	Dysarthria	Yes–No	[35]
29	Dizziness	Yes–No	[35]
30	Convulsion	Yes–No	[35]

### 3.4 Machine learning modeling

In various research in the field of stroke prediction, several algorithms were used to create models. In five articles, the RF algorithm was introduced as the best and most efficient algorithm for stroke prediction^21^, ^25^, ^29^, ^30^, ^32^ Two studies^8^, ^23^ recommended the SVM algorithm, and two other studies^22^, ^28^ chose the Stacking approach as the best approach for building ML algorithms. Two studies determined the XGBoost algorithm as the most efficient algorithm,^26^, ^37^ and in one study, the combination of XGBoost and RF algorithms was used as one of the algorithms of the new proposed model with high‐performance.^31^

**Figure 4:**
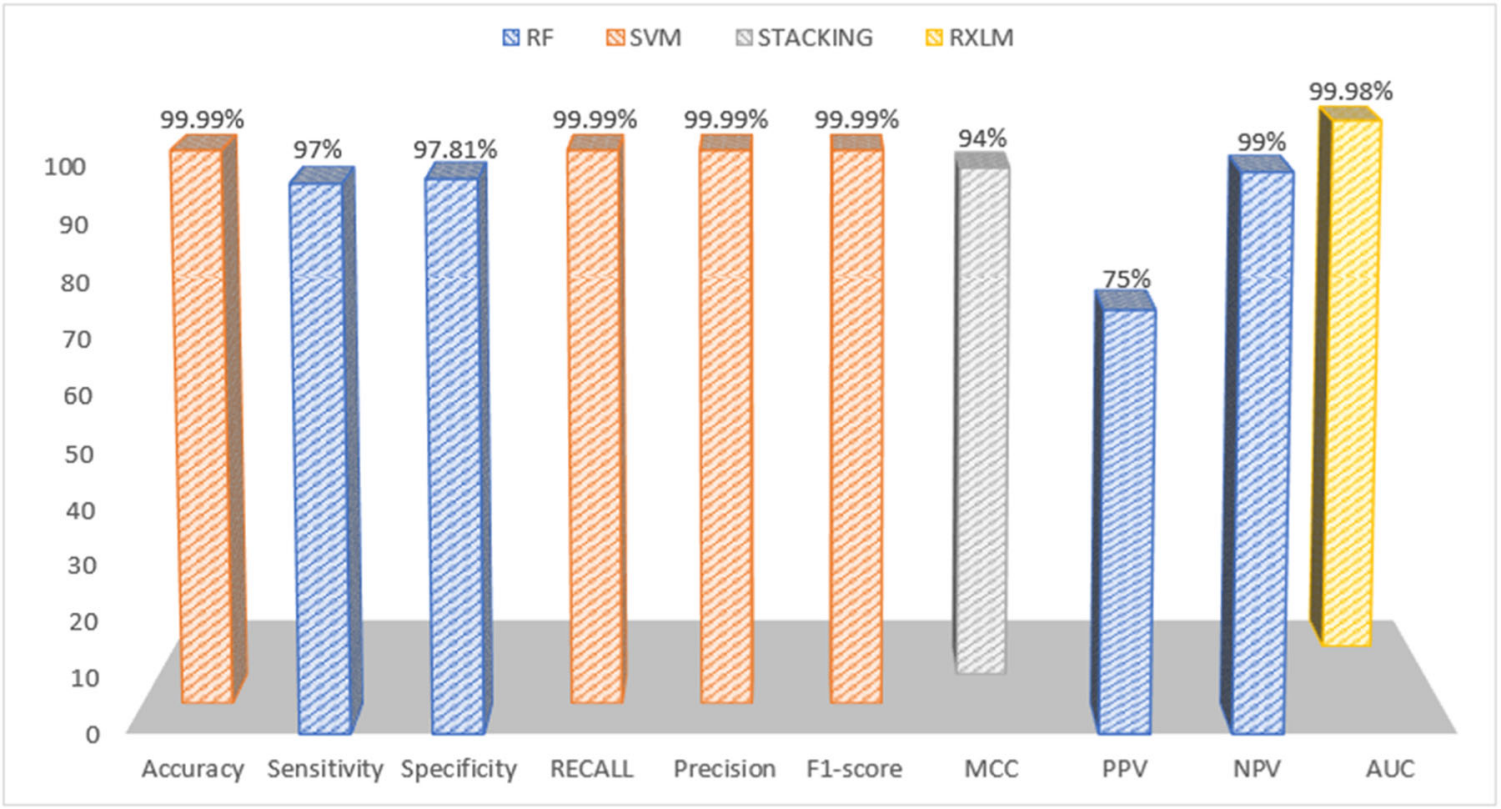
Comparison of the best performances recorded in different parameters of stroke prediction models.

However, a study that compared the performance of three algorithms (LR, RF, XGBoost) showed that all three algorithms obtained almost identical results with an accuracy of 65%.^35^ Another study that compared the performance of NB, SVM, RF, KNN, DT, Stacking, and Majority Voting algorithms showed that the performance of these algorithms was similar, except that the NB algorithm showed the lowest performance.^27^ The present study indicated that various algorithms such as RF, ANN, NB, SVM, DSGD, GBT&COX, Stacking, and XGBoost are the most efficient algorithms in this field, with a broad review of various studies in the stroke prediction area. (Figure 4).

### 3.5 Data pre‐processing

Data pre‐processing before developing a stroke prediction model is necessary and important to achieve maximum accuracy. Data preprocessing techniques are used to remove missing data, encoding labels, outliers, removing unwanted noise, etc. in the data set.^8^ A total of 11 articles used data pre‐processing techniques^8^, ^21^, ^22^, ^24^, ^27^, ^28^, ^30^, ^31^, ^32^, ^34^, ^37^ and 15 articles mentioned missing data.^8^, ^21^, ^22^, ^23^, ^24^, ^25^, ^26^, ^27^, ^28^, ^31^, ^32^, ^34^, ^35^, ^37^, ^39^ Seven articles mentioned the management of outlier data.^8^, ^21^, ^26^, ^31^, ^32^, ^34^, ^37^ A total of 25 different algorithms have been used in all studies, and RF (*N* = 16), LR (*N* = 13), and SVM (*N* = 12) algorithms were the most frequent (Table 4).

**Table 4:** Frequency of algorithms used in studies.

NO.	Algorithm	Number (n)	Frequency (%)
**1**	RF	16	14.54
**2**	LR	13	11.81
**3**	SVM	12	10.90
**4**	DSGD	1	0.91
**5**	RXLM	1	0.91
**6**	NB	11	10
**7**	COX & GBT	1	0.91
**8**	STACKING	4	3.64
**9**	ANN	2	1.82
**10**	XGBOOST	5	4.54
**11**	DT	11	10
**12**	BN	1	0.91
**13**	MLP	6	5.45
**14**	ADABOOST	3	2.73
**15**	RSF	1	0.91
**16**	KNN	9	8.18
**17**	SGD classifier	2	1.82
**18**	Extra trees	1	0.91
**19**	Voting classifier	3	2.73
**20**	RUSBoost	1	0.91
**21**	Majority Voting	2	1.82
**22**	FSRP	1	0.91
**23**	Gradient Boosting	1	0.91
**24**	Nearest Centroid	1	0.91
**25**	BRL	1	0.91
TOTAL		**110**	**100**

### 3.6 Data leakage

One of the less noticed problems in creating robust predictive models is data leakage. When data other than the training data are used in model development, data leakage occurs and the performance of the model decreases.^21^ To prevent data leakage, it is recommended to use Train‐test‐Split, that is, to separate the data into training data and test data. In Mridha,^21^ Alruily^31^ and Vodencarevic^23^ studies, they mentioned the problem of data leakage.

### 3.7 Handling imbalanced classes

Handling imbalanced classes is a common challenge in machine learning, particularly when dealing with classification problems. Imbalanced classes occur when one class (the majority class) has significantly more instances than the other class (the minority class). This imbalance can lead to biased models that perform poorly on the minority class.

Out of 20 studies reviewed, 14 studies of techniques COST, SMOTE, under‐sampling, Anomaly Detection Techniques, Ensemble Methods, and Random Over Sampling were used to solve the management of unbalanced classes, and 6 studies did not mention the techniques.

### 3.8 SMOTE

The SMOTE technique has been used in seven studies to solve unbalanced class management.^21^, ^22^, ^27^, ^28^, ^31^, ^32^, ^37^ In the method, SMOTE selects a minority class sample and finds its k‐nearest minority class neighbors. It then randomly selects one of these neighbors and generates a synthetic sample along the line segment joining the minority class sample and its neighbor.

### 3.9 Under‐sampling

In studies,^30^, ^33^, ^34^ the under‐sampling technique has been used to solve the management of unbalanced classes. In the technique Under‐sampling reduces the number of instances in the majority class to balance the class distribution.

### 3.10 Random over sampling

Random Over Sampling technique has been used in one study^8^ to solve imbalanced class management. Random over‐sampling involves randomly duplicating minority class samples to balance the class distribution. In the method ROS Randomly selects instances from the minority class and duplicates them until the classes are balanced.

### 3.11 Ensemble methods

In one study,^24^ the Ensemble Methods technique was used to solve the management of unbalanced classes. Ensemble Methods Uses multiple models to improve prediction accuracy for imbalanced datasets. Techniques like Balanced Random Forest are designed specifically for imbalanced data. Ensemble Methods Combines the predictions from several base models, such as in boosting or bagging and can improve overall model robustness and performance on the minority class.

### 3.12 Cost‐sensitive learning

Assigns a higher cost to misclassify minority class instances than majority class instances. The model is trained to minimize the total price, which can lead to better performance for the minority class. Cost‐sensitive Learning can Directly address the imbalance by penalizing errors on the minority class more heavily.

### 3.13 Anomaly detection techniques

This technique Treats the minority class as anomalies or outliers. Uses anomaly detection algorithms to identify and classify minority class instances and is effective when the minority class is very small and distinct from the majority class**.**

In one study^23^ four techniques, COST, SMOTE, under‐sampling, and Anomaly Detection Techniques were used for the balanced distribution of unbalanced classes.

### 3.14 Evaluation of models

Standard criteria have been used to evaluate the performance of models in the field of stroke prediction. In most of the included articles,^8^, ^21^, ^22^, ^26^, ^27^, ^28^, ^29^, ^32^, ^34^, ^37^, ^38^, ^39^ sensitivity, accuracy, specificity, AUC, recall, and F1‐score were used as criteria for evaluation. In addition to these criteria, in a number of papers,^24^, ^25^, ^28^, ^31^ Positive Predictive Value (PPV), Negative Predictive Value (NPV), Kappa, and Matthews Correlation Coefficient (MCC) were used to evaluate ML methods. These criteria have played an essential role in measuring the accuracy and efficiency of the models. Four articles used the Accuracy criterion,^30^, ^33^, ^35^, ^36^ and only one article used the AUC criterion.^23^ This diversity in the evaluation criteria shows the efforts of researchers to best match the criteria with different characteristics and cases in their studies. The diversity in evaluation criteria reflects the efforts to match the metrics to the characteristics and specific needs of each study. Table 5 shows the performance results of the best models.

**Table 5:** Results performance of best models.

Ref.	Best model	Accuracy	Sensitivity	Specificity	F1‐score	Precision	Recall	AUC	MCC	PPV	NPV
[8]	SVM	99.99	‐	‐	99.99	99.99	99.99	‐	‐	‐	‐
[21]	RF	90.36	‐	‐	91	88	94	‐	‐	‐	‐
[22]	Stacking	98	‐	‐	97.4	97.4	97.4	98.9	‐	‐	‐
[23]	SVM	‐	63	78	‐	‐	‐	70	‐	‐	‐
[24]	GBT & COX	Men:76 Women:80	Men:76 Women:67	Men:76 Women:81	‐	‐	‐	‐	‐	Men:26 Women:24	Men:97 Women:97
[25]	RF	96	97	96	‐	‐	‐	97	‐	75	99
[26]	XGBoost	84.78	‐	84.51	83.19	‐	85.12	92.2	‐	‐	‐
[27]	Stacking	96.74	‐	‐	98	97	100	‐	‐	‐	‐
[28]	Stacking	97	‐	‐	97	99	97	‐	94	‐	‐
[29]	RF	96.97	94.9	97.81	94.73	94.56	94.9	‐	‐	‐	‐
[30]	RF	98.42	‐	‐	99	100	98	‐	‐	‐	‐
[31]	RXLM (combination RF& XGBoost & LightGBM)	96.34	‐	‐	96.33	96.55	96.12	99.38	92.69	‐	‐
[32]	RF	96	‐	‐	96	97	95	‐	‐	‐	‐
[33]	ANN	75.02	‐	‐	‐	‐	‐	‐	‐	‐	‐
[34]	NB	82	‐	‐	82.3	79.2	85.7	‐	‐	‐	‐
[35]	LR RF XGBoost	65	‐	‐	‐	‐	‐	‐	‐	‐	‐
[36]	LR	‐	‐	‐	‐	‐	‐	79	‐	‐	‐
[37]	XGBoost	validating set:68 independent testing set:87	validating set:77 independent testing set:87	validating set:67 independent testing set:30	‐	‐	‐	validating set:78 independent testing set:83	‐	‐	‐
[38]	ANN	80	76.07	82.89	76.34	76.62	‐	‐	‐	‐	‐
[39]	DSGD	85.4	59.5	87.8	‐	‐	‐	87.5	‐	‐	‐

## 4 DISCUSSION

A stroke is a critical medical emergency that can be life‐threatening or cause irreversible damage. Hence, accurately diagnosing and preventing strokes is crucial. Currently, the application of ML algorithms in healthcare is rapidly increasing.^40^ These algorithms support physicians by leveraging their powerful processing capabilities for clinical decision‐making, prognosis, and, notably, forecasting the likelihood of a stroke. Unlike conventional prediction models that rely on calculations, ML models utilize various variables to accurately represent human physiology's complexities.^41^

In today's world, machine learning is emerging as a powerful tool in modeling complex and hidden relationships between clinical variables and physiology^42^ and traditional methods are not able to detect and predict stroke in the early stages. Consequently, a systematic review was conducted, focusing on articles that met specific research criteria. This review examined 20 articles to identify the most efficient ML algorithms for predicting strokes.

The review of all studies showed that ML has been an effective and positive method in predicting stroke, and most of the studies conducted in recent years indicate the use of ML in stroke prediction as an emerging tool in the healthcare area. In addition, among the articles reviewed in this research, most of them were conducted in Asian countries such as India, China, Saudi Arabia, Japan, Malaysia, Singapore, and Bangladesh, seeming to be due to the high rate of stroke in these countries and the importance of its prediction.

The articles included in the research had different data sets and modeling records, which showed the dynamics of ML models in predicting stroke. Moreover, the Kaggle data set was used more frequently in the studies. Matthew Chun et al. used the largest data set among the articles included in the study, which had 512,726 records.^24^ On the other hand, the smallest data set was in the study of Vodencarevic et al., who used the registry data set with 384 records.^23^

The research reviewed utilized a variety of algorithms to develop and present the model, employing different criteria to evaluate the ML algorithms' efficiency. In most studies (*n* = 17), accuracy served as a primary metric for gauging the effectiveness of the ML algorithms. Essentially, an algorithm's higher accuracy indicates superior performance and efficiency in predicting strokes, according to these studies.

In 10 studies, the accuracy of the stroke prediction algorithm was above 90%.^8^, ^21^, ^22^, ^25^, ^27^, ^28^, ^29^, ^30^, ^31^, ^32^ Among these 10 studies, five recommended the RF algorithm as the most efficient algorithm in stroke prediction.^21^, ^25^, ^29^, ^30^, ^32^ Although the RF algorithm has a high accuracy of 90 in all studies, the highest accuracy recorded was in the study of Biswas et al.^8^ in 2022 in Bangladesh, in which the SVM algorithm is the most efficient and best stroke prediction algorithm with an accuracy of 99%.

The variations in selecting the most efficient algorithms and their accuracy appear to stem from differences in sample size, data set, and data type. Consequently, further research must be conducted using consistent data sets, sample sizes, and data types to obtain more reliable outcomes and identify the most efficient ML algorithm model.

Choosing a suitable algorithm and data set can affect the performance of ML models in predicting stroke. Most articles used conventional ML algorithms to build prediction models, and a small number combined models to achieve more stable and robust models.

In some studies, using multiple models, combined methods, and the combination of these models in ML algorithms improved the accuracy of the final model compared to other models and approaches.^43^ For example, in the study of Alruily et al., the highest AUC value was achieved using the combination of XGBOOST, GBM, and RF models to improve the accuracy of their final “RXLM” model.^31^

Chun M et al.,^24^ in a study in 2021, found that when they combine Cox and GBT models, they have higher accuracy, specificity, and PPV for predicting stroke than when they use these models separately.

Among the 20 articles studied in our research, the highest degree of sensitivity is related to the study by Alanazi et al.^25^ and Xiao Zhang et al., in these studies, RF and XGB algorithms are known as the best models, respectively.

The swift advancement of artificial intelligence allows healthcare providers and decision‐makers to leverage ML models to pinpoint and understand risk factors associated with strokes. This aids in early prediction and minimizes the severe complications of strokes.^22^ In a 2021 study by Alanazi et al,^25^ using Random Forest (RF), Decision Tree (DT), Naive Bayes (NB), and Bayesian Network (BN) algorithms, Alanazi et al.^25^ demonstrated that nine laboratory tests, alongside age and gender, significantly correlate with stroke likelihood.

Ivanov et al.,^44^ emphasizing that data quality and pre‐processing play an important role in the development of reliable models, presented a detailed stroke data optimization model to improve stroke prediction, and in this the research of SVM algorithm with 98 percent accuracy and 97% recall has achieved a high score.

In the study of Dritsas et al.,^22^ the most critical and relevant risk factor for stroke is age. This is consistent with the findings of this systematic review, which confirms that the incidence of stroke after 45 years is twice as high, and 70% of all strokes occur after the age of 75.^45^

The findings of Sharma et al.^46^ study using a comparison of five algorithms (RF, JRip, NB, MLP, and DT) for early prediction of stroke showed that although lifestyle changes cannot prevent the inevitable occurrence of stroke, they can Significantly reduce the risk of stroke.

One of the challenges of machine learning in managing unbalanced classes is neglecting minority classes. The most popular technique used in the studies of this systematic review to manage unbalanced classes is the SMOTE technique, so that 35% of studies have suggested the use of the SMOTE technique. This technique helps in creating a more balanced data set without duplicating the minority class samples, leading to better generalization.

The under‐sampling technique has been used in studies^30^, ^33^, ^34^ to manage unbalanced classes, this technique Randomly removes instances from the majority class until the classes are balanced and this causes Simple to implement and reduces the size of the data set, making it faster to train models.

In the study of Biswas et al.,^8^ to manage unbalanced classes, the use of the Random Over‐sampling technique has been proposed and used, and the advantage of this technique is simple implementation and increasing the representation of the minority class.

In the study of Vodencarevic et al.,^23^ four techniques (COST, SMOTE, under‐sampling, and Anomaly Detection Techniques) were used for the balanced distribution of unbalanced classes.

Each of these techniques offers a way to improve model performance on imbalanced datasets, helping to ensure that the minority class is adequately represented and accurately predicted. The choice of technique depends on the specific characteristics of the data set and the problem being addressed.

In conducting this research, the authors encountered several limitations:

1. The study's scope is constrained by specific keywords and a defined time frame, with searches conducted exclusively in PubMed, Scopus, Web of Science, and IEEE databases. As a result, the search may only encompass some relevant studies.
2. The reporting of studies carried out in clinical settings appears to lack transparency, making the application of ML algorithms in these environments particularly challenging.
3. This research is limited to focusing solely on clinical and tabular data, excluding imaging data, in predicting stroke occurrences.

## 5 CONCLUSION

This research revealed that AI algorithms could help doctors and other healthcare professionals by predicting stroke across all examined texts. By moving beyond traditional methods that typically lack accuracy, are error‐prone, and consume considerable time and resources, the development of ML models enhances their accuracy and efficiency in disease prediction, including strokes. This advancement enables prompt interventions, potentially lowering the mortality rate and complications associated with strokes. However, despite the obvious advantages of ML algorithms over classical statistical approaches, there is a pressing need to establish standards and protocols to improve the accuracy and sensitivity of data modeling in ML.

## AUTHOR CONTRIBUTIONS

**Farkhondeh Asadi**: Conceptualization; Methodology. **Milad Rahimi**: Data curation; Formal analysis. **Amir Hossein Daeechini**: Writing—original draft; Writing—review and editing. **Atefeh Paghe**: Writing—original draft; Data curation.

## CONFLICT OF INTEREST STATEMENT

The authors declare no conflict of interest.

## ETHICS STATEMENT

This research was approved by the research ethics committee of Shahid Beheshti University of Medical Sciences with ethics code IR.SBMU.RETECH.REC.1402.849.

## TRANSPARENCY STATEMENT

The lead author Farkhondeh Asadi, Amir Hossein Daeechini affirms that this manuscript is an honest, accurate, and transparent account of the study being reported; that no important aspects of the study have been omitted; and that any discrepancies from the study as planned (and, if relevant, registered) have been explained.

## Data Availability

Data sharing is not applicable to this article as no new data were created or analyzed in this study. “All authors have read and approved the final version of the manuscript Farkhondeh Asadi had full access to all of the data in this study and take complete responsibility for the integrity of the data and the accuracy of the data analysis.”

## References

[1] Kuriakose D , Xiao Z . Pathophysiology and treatment of stroke: present status and future perspectives. Int J Mol Sci. 2020;21(20):7609. 33076218 10.3390/ijms21207609 PMC7589849

[2] Kim B , Schweighofer N , Haldar JP , Leahy RM , Winstein CJ . Corticospinal tract microstructure predicts distal arm motor improvements in chronic stroke. J Neurol Phys Ther. 2021;45(4):273‐281. 34269747 10.1097/NPT.0000000000000363 PMC8460613

[3] Pacchiano F , Tortora M , Criscuolo S , et al. Artificial intelligence applied in acute ischemic stroke: from child to elderly. Radiol Med (Torino). 2024;129(1):83‐92. 37878222 10.1007/s11547-023-01735-1 PMC10808481

[4] Harshitha KV , Harshitha P , Gupta G , Vaishak P , Prajna KB . Stroke prediction using machine learning algorithms. Int J Innov Res Engineer Manag. 2021;8:6‐9.

[5] Li Q , Chi L , Zhao W , et al. Machine learning prediction of motor function in chronic stroke patients: a systematic review and meta‐analysis. Front Neurol. 2023;14:1039794. 37388543 10.3389/fneur.2023.1039794 PMC10299899

[6] Huang R , Liu J , Wan TK , et al. Stroke mortality prediction based on ensemble learning and the combination of structured and textual data. Comput Biol Med. 2023;155:106176. 36805232 10.1016/j.compbiomed.2022.106176

[7] Kleindorfer DO , Towfighi A , Chaturvedi S , et al. 2021 guideline for the prevention of stroke in patients with stroke and transient ischemic attack: a guideline from the American Heart Association/American Stroke Association. Stroke. 2021;52(7):e364‐e467. 34024117 10.1161/STR.0000000000000375

[8] Biswas N , Uddin KMM , Rikta ST , Dey SK . A comparative analysis of machine learning classifiers for stroke prediction: a predictive analytics approach. Healthcare Analytics. 2022;2:100116.

[9] Lindsay P , Furie KL , Davis SM , Donnan GA , Norrving B . World Stroke Organization global stroke services guidelines and action plan. Int J Stroke. 2014;9Suppl A100:4‐13. 10.1111/ijs.12371 25250836

[10] Rahim AMA , Sunyoto A , Arief MR . Stroke prediction using machine learning method with extreme gradient boosting algorithm. MATRIK: Jurnal Manajemen, Teknik Informatika dan Rekayasa Komputer. 2022;21(3):595‐606.

[11] Xu Y , Ju L , Tong J , Zhou CM , Yang JJ . Machine learning algorithms for predicting the recurrence of stage IV colorectal cancer after tumor resection. Sci Rep. 2020;10(1):2519. 32054897 10.1038/s41598-020-59115-y PMC7220939

[12] Johnson KW , Torres Soto J , Glicksberg BS , et al. Artificial intelligence in cardiology. J Am Coll Cardiol. 2018;71(23):2668‐2679. 29880128 10.1016/j.jacc.2018.03.521

[13] Miceli G , Basso MG , Rizzo G , et al. Artificial intelligence in acute ischemic stroke subtypes according to toast classification: a comprehensive narrative review. Biomedicines. 2023;11(4):1138. 37189756 10.3390/biomedicines11041138 PMC10135701

[14] Bi Q , Goodman KE , Kaminsky J , et al. What is machine learning? A primer for the epidemiologist. Am J Epidemiol. 2019;188(12):2222‐2239. 31509183 10.1093/aje/kwz189

[15] Christodoulou E , Ma J , Collins GS , Steyerberg EW , Verbakel JY , Van Calster B . A systematic review shows no performance benefit of machine learning over logistic regression for clinical prediction models. J Clin Epidemiol. 2019;110:12‐22. 30763612 10.1016/j.jclinepi.2019.02.004

[16] Maadi M , Akbarzadeh Khorshidi H , Aickelin U . A review on human‐AI interaction in machine learning and insights for medical applications. Int J Environ Res Public Health. 2021;18(4):2121. 33671609 10.3390/ijerph18042121 PMC7926732

[17] Patel UK , Anwar A , Saleem S , et al. Artificial intelligence as an emerging technology in the current care of neurological disorders. J Neurol. 2021;268(5):1623‐1642. 31451912 10.1007/s00415-019-09518-3

[18] Soun JE , Chow DS , Nagamine M , et al. Artificial intelligence and acute stroke imaging. AJNR Am J Neuroradiol. 2021;42(1):2‐11. 33243898 10.3174/ajnr.A6883 PMC7814792

[19] Knight‐Greenfield A , Nario JJQ , Gupta A . Causes of acute stroke. Radiol Clin North Am. 2019;57(6):1093‐1108. 31582037 10.1016/j.rcl.2019.07.007 PMC7040961

[20] Boehme AK , Esenwa C , Elkind MSV . Stroke risk factors, genetics, and prevention. Circ Res. 2017;120(3):472‐495. 28154098 10.1161/CIRCRESAHA.116.308398 PMC5321635

[21] Mridha K , Ghimire S , Shin J , Aran A , Uddin MM , Mridha MF . Automated stroke prediction using machine learning: an explainable and exploratory study with a web application for early intervention. IEEE Access. 2023;11:52288‐52308.

[22] Dritsas E , Trigka M . Stroke risk prediction with machine learning techniques. Sensors. 2022;22:4670. 35808172 10.3390/s22134670 PMC9268898

[23] Vodencarevic A , Weingärtner M , Caro JJ , et al. Prediction of recurrent ischemic stroke using registry data and machine learning methods: the erlangen stroke registry. Stroke. 2022;53(7):2299‐2306. 35360927 10.1161/STROKEAHA.121.036557

[24] Chun M , Clarke R , Cairns BJ , et al. Stroke risk prediction using machine learning: a prospective cohort study of 0.5 million Chinese adults. J Am Med Inform Assoc. 2021;28(8):1719‐1727. 33969418 10.1093/jamia/ocab068 PMC8324240

[25] Alanazi EM , Abdou A , Luo J . Predicting risk of stroke from lab tests using machine learning algorithms: development and evaluation of prediction models. JMIR Format Res. 2021;5(12):e23440. 10.2196/23440 PMC8686476 34860663

[26] Yang Y , Zheng J , Du Z , Li Y , Cai Y . Accurate prediction of stroke for hypertensive patients based on medical big data and machine learning algorithms: retrospective study. JMIR Med Inform. 2021;9(11):e30277. 34757322 10.2196/30277 PMC8663532

[27] Alageel N , Alharbi R , Alharbi R , Alsayil M , Alharbi LA . Using machine learning algorithm as a method for improving stroke prediction. Int J Adv Computer Sci Appl. 2023;14:738‐744.

[28] Mostafa SA , Elzanfaly DS , Yakoub AE . A machine learning ensemble classifier for prediction of brain strokes. Int J Adv Computer Sci Appl. 2022;13(12):258‐266.

[29] Bandi V , Bhattacharyya D , Midhunchakkravarthy D . Prediction of brain stroke severity using machine learning. Revue d'Intelligence Artificielle. 2020;34:753‐761.

[30] Padimi V , Telu V , Ningombam DD . Performance analysis and comparison of various machine learning algorithms for early stroke prediction. ETRI J. 2022;45:1‐15.

[31] Alruily M , El‐Ghany SA , Mostafa AM , Ezz M , El‐Aziz AAA . A‐tuning ensemble machine learning technique for cerebral stroke prediction. Appl Sci. 2023;13:5047.

[32] Tazin T , Alam MN , Dola NN , Bari MS , Bourouis S , Monirujjaman Khan M . Stroke disease detection and prediction using robust learning approaches. J Healthc Eng. 2021;2021:1‐12. 10.1155/2021/7633381 PMC8641997 34868531

[33] Nwosu CS , Dev S , Bhardwaj P , et al. Predicting stroke from electronic health records. Annu Int Conf IEEE Eng Med Biol Soc. 2019;2019:5704‐5707. 31947147 10.1109/EMBC.2019.8857234

[34] Sailasya G , Kumari GLA . Analyzing the performance of stroke prediction using ML classification algorithms. Int J Adv Computer Sci Appl. 2021;12:539‐545.

[35] Uchida K , Kouno J , Yoshimura S , et al. Development of machine learning models to predict probabilities and types of stroke at prehospital stage: the Japan Urgent Stroke Triage Score Using Machine Learning (JUST‐ML). Transl Stroke Res. 2022;13(3):370‐381. 34389965 10.1007/s12975-021-00937-x PMC9046322

[36] Wang Q , Zhang L , Li Y , Tang X , Yao Y , Fang Q . Development of stroke predictive model in community‐dwelling population: a longitudinal cohort study in Southeast China. Front Aging Neurosci. 2022;14:1036215. 36620776 10.3389/fnagi.2022.1036215 PMC9813513

[37] Zhang X , Fei N , Zhang X , Wang Q , Fang Z . Machine learning prediction models for postoperative stroke in elderly patients: analyses of the MIMIC database. Front Aging Neurosci. 2022;14:897611. 35923545 10.3389/fnagi.2022.897611 PMC9341133

[38] Hassan FH , Omar MA . Recurrent stroke prediction using machine learning algorithms with clinical public datasets: an empirical performance evaluation. Baghdad Sci J. 2021;18(4 suppl):1406.

[39] Penafiel S , Baloian N , Sanson H , et al. Predicting stroke risk with an interpretable classifier. IEEE Access. 2020;9:1154‐1166.

[40] Srinivasa KG , Siddesh GM , Manisekhar S . Statistical modelling and machine learning principles for bioinformatics techniques, tools, and applications. Springer Nature; 2020.

[41] Li X , Chen Z , Jiao H , et al. Machine learning in the prediction of post‐stroke cognitive impairment: a systematic review and meta‐analysis. Front Neurol. 2023;14:1211733. 37602236 10.3389/fneur.2023.1211733 PMC10434510

[42] Chahine Y , Magoon MJ , Maidu B , del Álamo JC , Boyle PM , Akoum N . Machine learning and the conundrum of stroke risk prediction. Arrhythm Electrophysiol Rev. 2023;12:e07. 37427297 10.15420/aer.2022.34 PMC10326666

[43] de Groof AJ , Struyvenberg MR , van der Putten J , et al. Deep‐learning system detects neoplasia in patients with Barrett's esophagus with higher accuracy than endoscopists in a multistep training and validation study with benchmarking. Gastroenterology. 2020;158(4):915‐929.e4. 31759929 10.1053/j.gastro.2019.11.030

[44] Ivanov IG , Kumchev Y , Hooper VJ . An optimization precise model of stroke data to improve stroke prediction. Algorithms. 2023;16(9):417.

[45] Benjamin EJ , Muntner P , Alonso A , et al. Heart disease and stroke Statistics‐2019 update: a report from the American Heart Association. Circulation. 2019;139(10):e56‐e528. 30700139 10.1161/CIR.0000000000000659

[46] Sharma C , Sharma S , Kumar M . Early stroke prediction using machine learning. 2022. International Conference on Decision Aid Sciences and Applications (DASA); 2022 23‐25 March 2022.

